# A case of iridoschisis with partial lens dislocation in both eyes

**DOI:** 10.1186/s12886-024-03330-y

**Published:** 2024-02-14

**Authors:** Tong-Tong Niu, Wen-Jian Xin

**Affiliations:** Department of Ophthalmology, Xinjiang 474 Hospital, Urumchi, 830000 China

**Keywords:** Iridoschisis, Iris degeneration, Phacoemulsification, Lens dislocation

## Abstract

**Background:**

Iridoschisis is a rare condition that primarily affects individuals aged 60–70 years. The predominant characteristics of iridoschisis involve the tissue splitting and separation of the iris stromal layers, often resulting in two distinct layers and the presence of floating fibers in the anterior chamber. This article reports the case of a 48-year-old male with iridoschisis with partial lens dislocation in both eyes.

**Case presentation:**

Trauma is the leading factor in the development of iridoschisis. However, there is no documented case of ocular trauma in the patient’s medical history. Visible white atrophic fibers were observed bilaterally in the anterior iris stroma of both eyes of the individual, accompanied by a small quantity of iris tissue within the anterior chamber. In this instance, the magnitude of the iridoschisis corresponded with the degree of lens dislocation. We were apprised that the patient had regularly used a cervical massager for a prolonged period of time, positioning it upon the ocular region. Frequent stimulation of both eyes with excessive force resulted in the development of iridoschisis and the partial dislocation of the lens.During the initial surgical procedure, phacoemulsification (Phaco) was carried out on the left eye without the placement of an intraocular lens (IOL). Following a two-month interval, we proceeded with the IOL suspension. Subsequently, the right eye underwent Phaco, accompanied by the implantation of an IOL. After closely monitoring the patient’s progress for two months, it was evident that their vision had significantly improved, substantiating the success of the surgical interventions.

**Conclusions:**

This finding posits that the recurrent friction applied to both eyes may induce iridoschisis and various ocular complications. In the event of ocular intricacies manifesting, expeditious medical intervention becomes imperative.

## Background

In 1922, Schmitt [1] initially described iridoschisis as a rare disorder. Then, Loewnstein [2] in 1945 redefined it as a separation of the anterior iris stroma from the posterior iris stroma and the muscular layer. More specifically, this health condition is frequently observed in those who are over the age of 60, often coinciding with cataracts and closed-angle glaucoma [3–6]. The condition’s rarity may play a role in the scarcity of epidemiological data and research.

Proper management of iridoschisis requires addressing the resulting complications, particularly when it progresses to stages with corneal endothelial loss. In iridoschisis, the anterior iris stroma separates into numerous pigmented and white atrophic fibers. One end of these fibers is attached to the iris proper, while the other floats in the anterior chamber. The contact between the floating iris fibers and the corneal endothelium can result in corneal endothelial edema and, in severe cases, corneal decompensation. Treating such corneal edema through corneal transplantation, such as penetrating corneal transplantation or posterior elastic lamellar endothelial corneal transplantation, has been shown to be highly effective [7, 8]. Additionally, changes resembling pigmentation may be observed in the posterior iris stroma [8]. Factors such as the patient’s overall eye health, the presence of concurrent ocular conditions like cataracts and glaucoma, and the success of surgical interventions may influence the long-term prognosis. A noteworthy case involves a 48-year-old male who presented with bilateral iridoschisis and partial lens dislocation, a rare occurrence. The specifics of this case are outlined as follows.

### Case presentation

The 48-year-old male patient was admitted to the ophthalmology department for ongoing binocular visual acuity issues that have persisted for over a year, along with a significant decline in vision over the past month. According to the patient, there were instances of discomfort in both eyes, characterized by dryness and photophobia, without any accompanying signs of redness or pain in the eyes.The ocular assessment revealed that the patient’s uncorrected visual acuity was 20/80 in the right eye and 20/300 in the left eye. Despite attempts to correct it, there was no improvement in visual acuity. Using a Goldmann applanation tonometer, the patient’s intraocular pressure (IOP) was measured to be 14 mmHg in the right eye and 13 mmHg in the left eye (1 mmHg = 0.133 Kpa).

**Fig. 1:**
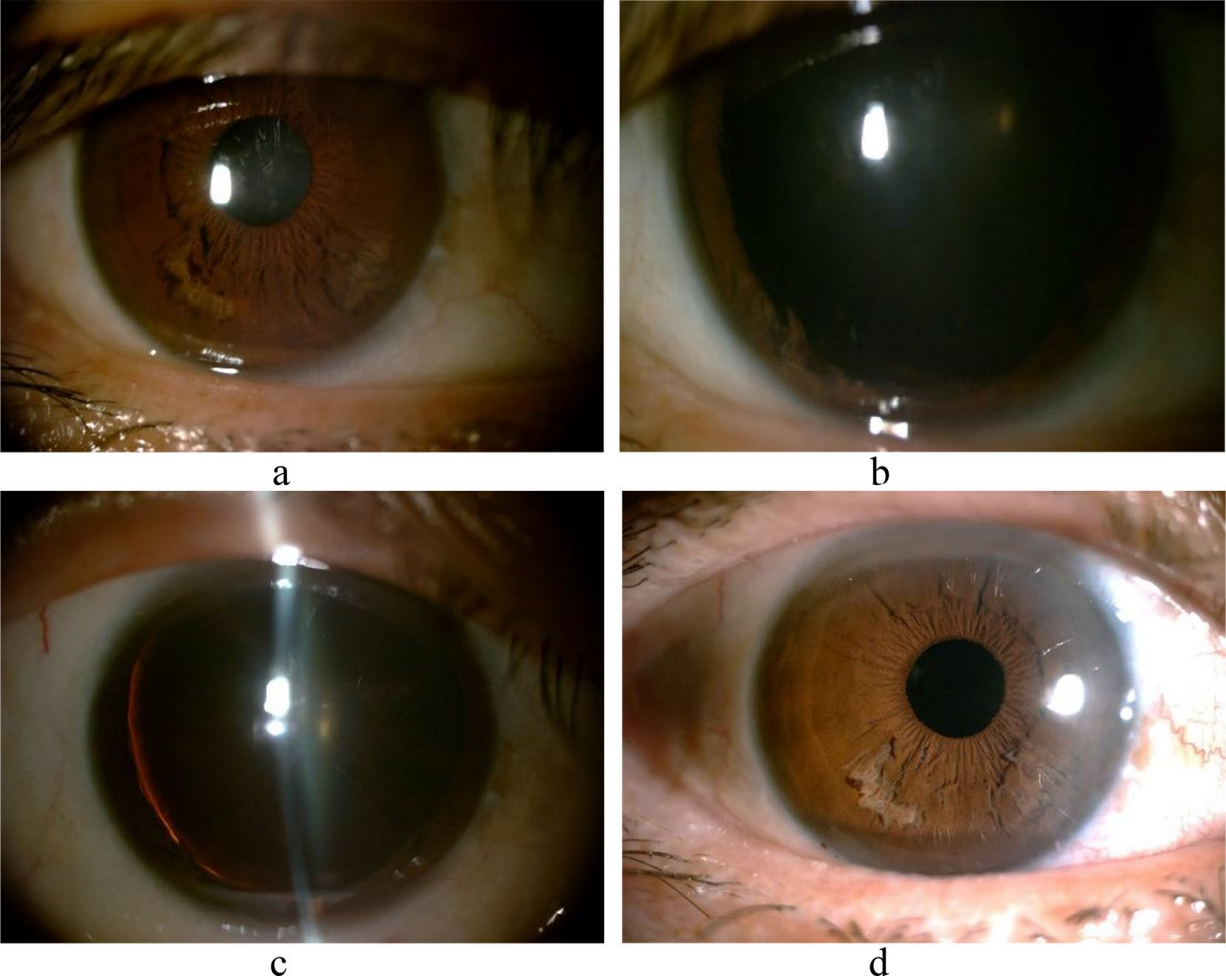
Photograph of the anterior chamber of the right eye. **a**: Iridoschisis in the right eye, temporally and nasally; **b** & **c**: After pupil dilation, the iris of the right eye is curled temporally, the split iris is floating in the atrial fluid, the suspensory ligament is disarticulated at points 1–6, and the lens is partially dislocated; **d**: Two months postoperatively, the IOL is in the correct position and the iris splitting is not enlarged

A slit-lamp examination identified iridoschisis in the right eye, specifically in the subnasal and temporal regions (Fig. 1a). This was evidenced by the visibility of white atrophic fibers visible in the anterior stroma of the iris, as well as a small amount of free iris tissue in the anterior chamber. The atrophied iris fibers were discovered to be bent toward the front, with a portion of the iris fibers still attached around the iris and ciliary body while others floated in the anterior chamber, resembling seaweed. The suspensory ligaments were disarticulated at points 1–6, causing a partial dislocation of the lens (Fig. 1b & c). Based on the Lens opacities classification system ll (LOCS ll) [9], the clouding of the patient’s right eye lens was graded as C4N3P2.

**Fig. 2:**
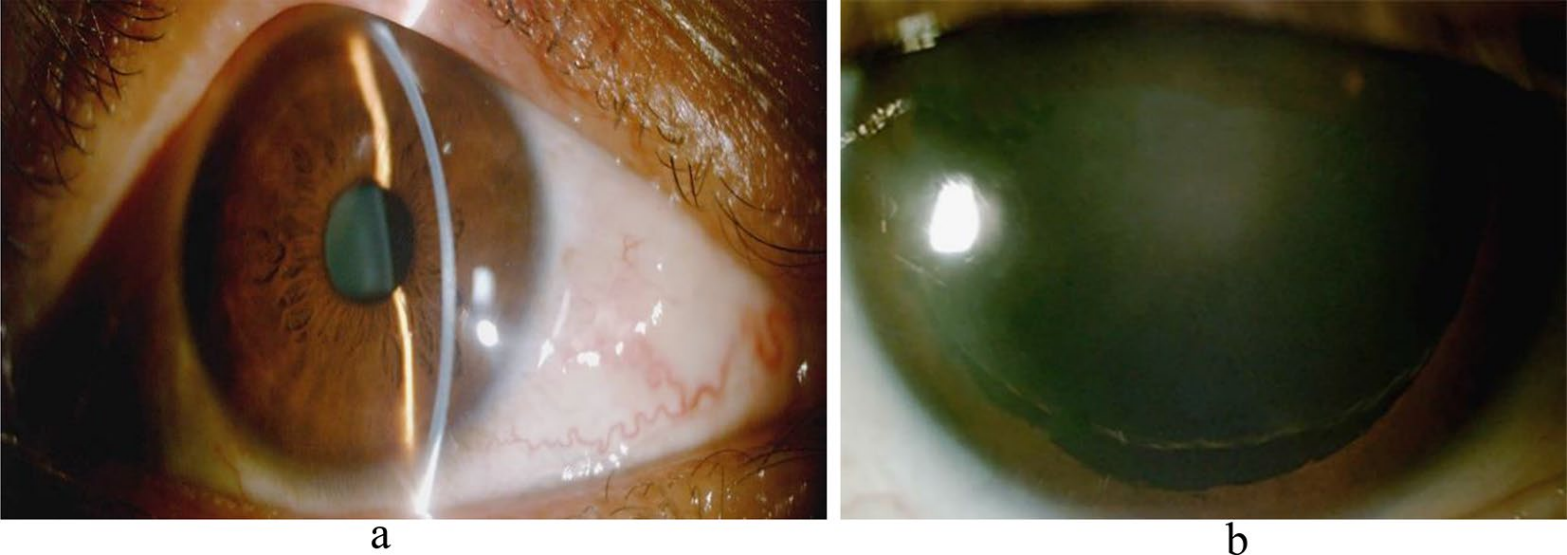
Photograph of the anterior segment of the left eye. **a**: Splitting is visible below the iris of the left eye; **b**: After pupil dilation, the suspensory ligament of the left eye is disarticulated at points 1–10 and the lens is partially dislocated

The lower part of the left eye exhibited iridoschisis, along with a small amount of free iris tissue in the anterior chamber, dislocation of the suspensory ligaments at points 1–10, partial dislocation of the lens (Fig. 2a & b), lens clouding, and the presence of C3N3P2. The presence of considerable lens opacity in both eyes prevented a comprehensive analysis of fundus deconstruction and optic disc. Additionally, the patient’s poor visual acuity rendered the visual field findings inconclusive. The patient’s 24-hour IOP was monitored and fluctuated between 13 and 15 mmHg. In terms of axial lengths, the right eye was 25.39 mm and the left eye was 25.45 mm.

**Fig. 3:**
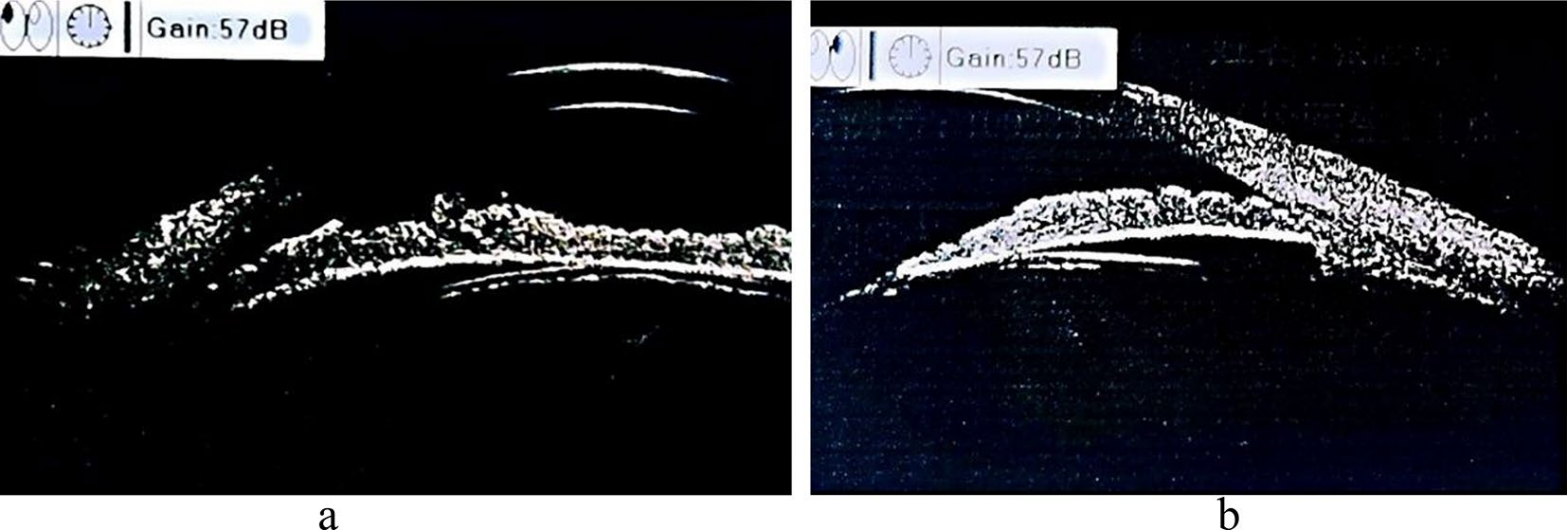
Ultrasound biomicroscopy with iris root splitting in both eyes. **a**: Right eye; **b**: Left eye

The endothelial cell density (ECD) of the right cornea measured 2946/mm2, exhibiting a hexagonal cell percentage (HEX) of 48% and a 27% coefficient of variation (CV). Meanwhile, in the left cornea, the ECD was 2855/mm2, with a HEX of 47% and a CV of 23%. The patient’s ocular ultrasound findings indicated the presence of cataracts and posterior scleral staphyloma in both eyes. Additionally, the ultrasound biomicroscopy (UBM) revealed iris root splitting in both eyes, anterior displacement of the ciliary body, disarticulation of the suspensory ligament of the crystalline lens beneath both eyes, and iris damage underneath the eyes (Fig. 3a & b). The patient had no record of pre-existing illnesses, previous ocular procedures, ocular trauma, or hereditary ocular diseases. Their rapid plasma regain (RPR) test for syphilis was negative

Due to the patient’s impaired vision in the left eye, they requested that it be treated first. We performed phacoemulsification (Phaco) in the left eye under local anesthesia. Considering the significant lens dislocation in the left eye, an intraocular lens (IOL) was not implanted and an IOL (HOYA PY-60AD, Japan) suspension surgery was performed in the left eye one month post-Phaco surgery. Two months post-surgery, another eye examination was carried out. The extent of iris atrophy remained unchanged, the patient’s best-corrected visual acuity was 20/40, and the intraocular pressure in the left eye was 11 mmHg with an ECD of 2393/mm2, a HEX of 35%, and aCV of 22%.

The patient’s left eye surgery was followed by Phaco surgery on their right eye after two months, in order to manage the cataract. The surgery was performed under local anesthesia, and an IOL (HOYA-PY60AD, Japan) was inserted. During the procedure, we utilized an iris retractor to stabilize the suspensory ligament and took measures to place capsular bag tension loops, ultimately resulting in the successful implantation of a satisfactory IOL. As part of the post-surgery treatment, the patient was routinely prescribed anti-inflammatory, anti-infective, and dilating eye drops.

At the 2-month follow-up, it was observed that the patient displayed no growth in the extent of the small number of white atrophic fibers on the iris of their right eye (Fig. 1d). The positive outcome was ensured by optimally positioning the IOL. The patient’s best-corrected visual acuity was 20/25, and the IOP in the right eye was 14 mmHg with an ECD of 2278/mm2, a HEX of 43%, and a CV of 22%.

## Discussion

Iridoschisis is a rare disorder that typically affects individuals in the 60–70 age range. Although iridoschisis has been previously reported in the literature to be an age-related condition (as it often coincides with cataracts), there is no conclusive evidence of a direct age correlation. In most cases, iridoschisis manifests as the splitting of the tissue and separation of the iris stromal layers (usually splitting into two layers) with fibers floating in the anterior chamber. Once the anterior stroma splits, the posterior stroma undergoes shrinkage and thinning, ultimately attaching to the iris stroma proper. Although the etiology of iridoschisis is not fully established, trauma, syphilis, and congenital developmental abnormalities may play a role [6]. Current consensus suggests that the pathogenesis of the disease is physiologic atrophy of the iris stroma layer, followed by degenerative changes, tearing, and pulling within the stroma. Subsequently, lumen enlargement and the formation of clefts take place. Electron microscopy studies have revealed marked thinning of the stroma in the affected regions and a decline in the amount of collagen fibers, while the stromal vessels and nerves retain a normal appearance [10].

The presence of secondary glaucoma is frequently observed in those with iridoschisis [11]. The finely divided iris fibrous tissue obstructs the angle of the anterior chamber. As a result, the outflow of aqueous humor is impeded, thus frequently causing secondary open-angle glaucoma. Several theories suggest that iridoschisis may be secondary to closed-angle glaucoma, which could develop due to the intermittent or persistent elevation of intraocular pressure (IOP).

Iridoschisis may manifest prior to the closure of the atrial angle (i.e., iridoschisis occurs first, followed by a narrowing or even closure of the atrial angle), leading to an elevated IOP [11]. The patient in this study had relatively low intraocular pressure, no visible pigmentation via angle gonioscopy, and an open atrial angle. Therefore, it was postulated that no secondary glaucoma was detected at the time. Previous studies have shown [8] that iridoschisis can coincide with corneal endothelial dystrophy, as a result of floating debris from the iris stromal making contact with the corneal endothelium.

Corneal edema is the accumulation of fluid in the corneal tissue, leading to swelling. Irritation occurs, and localized corneal edema is triggered when the floating iris fibers come into contact with the corneal endothelium in this particular case. The corneal endothelium, located on the inner surface of the cornea, plays a vital role in maintaining corneal transparency by regulating fluid balance. Endothelial cell count can decrease due to recurrent irritation and localized edema. The corneal endothelium plays a critical role in preserving the clarity of the cornea by actively pumping out excess fluid. A decline in endothelial cells can ultimately result in corneal endothelial decompensation. Corneal decompensation occurs when the corneal endothelium is unable to maintain the appropriate balance of fluid, leading to a loss of corneal transparency. This loss of transparency can significantly impact vision, resulting in visual impairment. In summary, the separation of the anterior iris stroma in this condition can trigger a chain of events. These complications include localized corneal edema, irritation of the corneal endothelium, reduction in the number of endothelial cells, and, ultimately, corneal endothelial decompensation, leading to vision loss. Meanwhile, the corneal endothelial cell counts in both eyes of our patient were found to be within the normal range.

The occurrence of familial iridoschisis with secondary glaucoma has led some experts to propose a potential hereditary link to this disorder, thus recommending that individuals with affected family members undergo eye examinations [6]. In this case, the patient’s immediate family members underwent detailed ocular examinations, and no indications of iridoschisis were observed. It should be noted that iridoschisis has been linked to lens subluxation in previous studies [4]. In instances like these, lens subluxation creates friction against the iris, thereby causing iridoschisis. An earlier study [5] detailed a distinct occurrence of bilateral iridoschisis coexisting with keratoconus and severe allergic ophthalmopathy. The coexistence of these symptoms was attributed to the patient’s long-term habitual eye rubbing and severe allergic ophthalmopathy. The patient was found to be right-handed in this specific situation, with a more pronounced occurrence of keratoconus in the right eye in comparison to the left eye. The repeated and forceful rubbing of the eyes, most likely caused by severe allergic ophthalmopathy, is the underlying mechanism for this association. The friction between the lens subluxation and the iris may contribute to the development of iridoschisis. It is worth noting that habitual eye rubbing can negatively impact ocular structures, and in this particular scenario, it likely influenced the manifestation of bilateral iridoschisis alongside keratoconus. Adler [12] reported a case involving iridoschisis in the right eye and lens subluxation in both eyes. Previous ocular eczema in the patient was considered a potential factor in the development of iridoschisis and lens subluxation, possibly due to prolonged eye rubbing. Ocular eczema is characterized by inflammation of the eyelids and the surrounding skin, often accompanied by itching. Extended periods of eye rubbing, especially when accompanied by ocular eczema, can exacerbate the mechanical stress on ocular structures, potentially resulting in conditions like iridoschisis and lens subluxation. It is important to highlight that the specifics of each individual case may vary, and the relationship between ocular conditions like iridoschisis and lens subluxation, and eye rubbing can be complex. Nevertheless, in the reported case, the history of ocular eczema and the bilateral involvement seen in the case suggest a potential link between prolonged eye rubbing and the onset of iridoschisis and lens subluxation.

The management of iridoschisis involves regular observation for any complications from coexisting ocular conditions, such as cataracts or glaucoma. In cases complicated by cataracts, phacoemulsification is required. If secondary glaucoma is detected, an iris iridotomy or trabeculectomy may be necessary, depending on the degree of closure of the aperture angle. As the condition advances to the point of corneal endothelial loss, staged surgical interventions can be performed. The standard protocol consists of a sequence of actions, starting with cataract extraction and superficial iridectomy performed using an anterior vitrector device, followed by the subsequent step of posterior elastic lamina endothelial corneal transplantation [8]. In our case, the patient presented with diminished visual acuity in their left eye and requested surgical treatment of the left eye first. We performed Phaco on the left eye. The dislocation was too extensive to allow for the implantation of an IOL in the initial stage. Consequently, IOL implantation was performed during the second stage. The right eye was also subjected to Phaco and IOL implantation.

In this case, the extent of the iris dehiscence was consistent with the extent of the lens dislocation. Hence, our initial investigation focused on determining if the ocular symptoms were associated with ocular trauma. However, the patient denied any past occurrences of ocular trauma. Following the surgical procedure, the patient disclosed to us that he had utilized a neck massager on both of their eyes for six consecutive months. After engaging in a discussion, we concluded that the repetitive mechanical friction and stimulation must likely caused the suspensory ligament to disassociate, leading to the partial dislocation of the lens. The dislocated lens continually irritated the iris, leading to a separation between the anterior and posterior iris stroma and ultimately causing secondary iris splitting.

## Conclusions

This case underscores that frequent rubbing of both eyes may lead to iridoschisis and other ocular complications. Timely medical intervention is crucial when dealing with ocular complications. Equally important, the use of neck massagers on the eyes can lead to serious complications and should therefore be avoided. In conclusion, individuals should refrain from using devices in ways that deviate from their intended purpose, as this can ultimately result in harm to themselves.

## Data Availability

The datasets presented in this study is available from the corresponding author upon request.
